# Spatiotemporal Expression and Haplotypes Identification of *KRT84* Gene and Their Association with Wool Traits in Gansu Alpine Fine-Wool Sheep

**DOI:** 10.3390/genes15020248

**Published:** 2024-02-16

**Authors:** Xueqin Yu, Shaobin Li, Huitong Zhou, Fangfang Zhao, Jiang Hu, Jiqing Wang, Xiu Liu, Mingna Li, Zhidong Zhao, Zhiyun Hao, Bingang Shi, Jon G. H. Hickford

**Affiliations:** 1 Gansu Key Laboratory of Herbivorous Animal Biotechnology, Faculty of Animal Science and Technology, Gansu Agricultural University, Lanzhou 730070, China; yuxq@st.gsau.edu.cn (X.Y.); zhaofangfang@gsau.edu.cn (F.Z.); huj@gsau.edu.cn (J.H.); wangjq@gsau.edu.cn (J.W.); liuxiu@gsau.edu.cn (X.L.); limn@gsau.edu.cn (M.L.); zhaozd@gsau.edu.cn (Z.Z.); haozy@gsau.edu.cn (Z.H.); shibg@st.gsau.edu.cn (B.S.); 2 International Wool Research Institute, Gansu Agricultural University, Lanzhou 730070, China; zhouh@lincoln.ac.nz; 3 Gene-Marker Laboratory, Faculty of Agricultural and Life Sciences, Lincoln University, Lincoln 7647, New Zealand

**Keywords:** Gansu Alpine Fine-wool sheep, keratin, SNPs, spatiotemporal expression

## Abstract

Keratin (K) is a major protein component of hair and is involved in hair growth and development. In this study, we analysed the expression, localization, and polymorphism of the K84 gene (*KRT84*) in Gansu Alpine Fine-wool sheep using immunofluorescence, RT-qPCR, and PARMS (penta-primer amplification refractory mutation system). Haplotypes of *KRT84* were also constructed and their relationship with wool traits analysed. It was revealed that *KRT84* was highly expressed in hair follicles, including the inner root sheath, outer root sheath, and hair medulla and at all six lamb ages investigated from 1 to 270 days of age. Three SNPs were detected in *KRT84* exon 1, and they formed three haplotypes (named *H1*, *H2,* and *H3*) and six genotypes. Analyses revealed an association between haplotype combinations (diplotypes) and the mean fibre curvature, mean staple length, mean staple strength, mean fibre diameter, the coefficient of variation of fibre diameter, and comfort factor for these sheep. These results suggest that *KRT84* is of importance in determining several key traits in Gansu Alpine Fine-wool sheep and that the gene could possibly be used as a genetic marker for wool trait selection in these sheep.

## 1. Introduction

Keratinous hair is unique to mammalian skin. It is a terminally differentiated tissue that forms in hair follicles and grows from the inner to the outer part of the epidermis [1]. The hair follicle contains both mesenchymal and epithelial components, separated by a basement membrane. In the follicle bulb, the dermal papilla (DP) consists of mesenchymal cells that function in the regulation of hair growth. The DP contains blood vessels and nerve endings that supply oxygen and nutrients to the root of the hair and is associated with the epithelial progenitor populations of the follicle. The bulb produces several distinct cell layers, including the outer root sheath, the inner root sheath, and the hair shaft [2]. Maria et al. [3] divided the process of hair follicle development into three phases: follicular substrate formation, organogenesis, and cell differentiation, whereas Saxena et al. [4] divided the stages of hair follicle development in more detail, based on the differentiation of different types of cells within the follicle.

Keratins (Ks) are fibrous proteins found in various tissues. They typically serve a protective function, being found in a variety of animal hairs, hoof tissues, feathers, and scales, and they are the major structural proteins in hair follicle cells. In sheep, keratins are produced in the wool follicle, and they are intrinsically involved in determining the structural characteristics of wool fibres. Accordingly, keratin genes are candidate genes for determining variation in wool fibre characteristics and biology [5]. In wool, keratin proteins form complex structures through the interaction of their amino acid side chains with other proteins. Of these interactions, the formation of strong covalent disulfide bonds is prominent [6]. While the internal structures of different mammalian hairs are similar, there are notable differences in morphology, with these in part attributable to variation in the keratin proteins and their molecular variability [7].

The keratins can be classified as epithelial keratins and hair keratins, and they have been divided into type I and type II families based on their sites of expression and functions. These keratin families are differentially expressed during hair fibre development [5,8]. Type I keratin genes (*KRTs*) are 4 to 5 kilobase pairs in length and have six introns, while the type II *KRTs* are larger, ranging from 7 to 9 kilobase pairs in length with eight introns. McLaren et al. [9] used linkage analyses to reveal that the type I and type II genes are located on chromosomes 11 and 3, respectively, in sheep, and both types of proteins are encoded by multiple genes [9,10].

Of the different keratins, K84 has been revealed to be expressed at high levels in plucked hairs collected from domestic horses (*Equus ferus caballus*) [11]. In cashmere goats (*Capra hircus*), it was found that *KRT84* promoter activity can be affected by *HOXC13* [12], while in sheep (*Ovis aries*), it was revealed that *KRT84* transcripts were present only in the fibrous cuticle, and that this may have resulted in a 3:2 ratio of type I to type II in the epibulbar region of the follicle, compared to a ratio of 2:1 in humans [13]. To date, there are no reports on the role of *KRT84* in wool follicle growth and development or whether the gene affects wool traits. To explore *KRT84*’s role in wool production in Gansu Alpine Fine-wool sheep from China, the spatiotemporal expression of *KRT84* was investigated, sequence variation was revealed in the gene, and haplotypes of the gene were tested to ascertain if they were associated with variation in key wool traits.

## 2. Materials and Methods

### 2.1. Sheep and Sampling

Two hundred and twenty-five Gansu Alpine Fine-wool sheep ewes of around three years in age with the same feeding levels and living environment were selected for the penta-primer amplification refractory mutation system (PARMS) and genetic association analyses. Blood was collected from the jugular vein of each sheep using a 5 mL sodium heparin tube, numbered, and stored at −20 °C until needed. Wool samples from the mid-flank of each sheep were collected by clipping against the skin and sealed in self-sealing bags with identification details. The mean fibre diameter (MFD), fibre diameter standard deviation (FDSD), coefficient of variation of fibre diameter (CVFD), mean staple length (MSL), mean fibre curvature (MFC), comfort factor (CF), and mean staple strength (MSS) for each wool sample were assessed. All wool testing was performed by New Zealand Pastoral Measurements LTD (Ahuriri, Napier, New Zealand) using International Wool Textile Organisation (IWTO; https://iwto.org/)-endorsed methods (https://iwto.org/resources/wool-testing-resources/; accessed on 15 September 2023).

Three new-born Gansu Alpine Fine-wool ewe lambs were selected for the immunofluorescence analysis. Skin tissues were collected from a 5 cm^2^ area of the posterior border of their scapulae at 1 day (d), 30 d, 60 d, 90 d, 180 d, and 270 d of age, using a skin sampler (0.88 cm in diameter). The skin samples were collected after localized injection of lidocaine hydrochloride (Jiangxi Roaching Longboat Veterinary Medicine Co., Ltd., Nanchang, China) at the site of collection for anaesthesia, and penicillin (Suicheng Pharmaceutical Co., Ltd., Zhengzhou, China) and Yunnan Baiyao (Yunnan Baiyao Group Co., Ltd., Kunming, China) were applied to the wound after the sampling to prevent infections.

After being cleaned with diethylpyrocarbonate-treated water, a portion of the skin tissue was placed in a tube containing 4% paraformaldehyde and stored at 4 °C. Separate portions of the skin samples were also placed in tubes containing an RNA protective solution (Servicebio Biotechnology Co., Ltd., Wuhan, China), which was allowed to penetrate the tissue to stabilize and protect the cellular RNA. These samples were stored in liquid nitrogen.

### 2.2. DNA Extraction

DNA was extracted using blood genomic DNA extraction kits (Tiangen Biochemical Technology Co., Ltd., Beijing, China). After the DNA was extracted, it was stored in TE buffer and quantified using a NanoDropTM 2000 spectrophotometer (ThermoFisher, Shanghai, China). The quality of the DNA was checked with agarose gel electrophoresis, and it was stored at −20 °C for subsequent analysis.

### 2.3. RNA Extraction

RNA was extracted from the skin tissue following the instructions provided with the Trizol reagent (Shanghai Yuanyye Bio-Technology Co., Ltd., Shanghai, China). The concentration of the extracted RNA was determined using ultraviolet spectrophotometry and the RNA samples were stored at −80 °C until needed.

### 2.4. Detecting KRT84 Sequence Variaiton

Based on the sequence variation reported for the *KRT84* reference sequence ENSOARG00020013379 in the Ensembl database (https://www.ensembl.org), exon 1 was chosen for sequence variation analysis. Primers were designed using Oligo 7 software (https://www.oligo.net/) to amplify the entire exon 1 coding sequence. The PCR primer sequences are shown in Table 1.

The PCR amplifications were carried out in a 20 μL reaction, containing 100 ng genomic DNA, 0.5 U of Taq DNA polymerase (Takara, Dalian, China), 2.5 mM Mg^2+^, 150 μM of each dNTP (Takara), and 0.25 μM of each primer. The thermal cycle parameters for amplification consisted of an initial denaturation incubation at 95 °C for 2 min, followed by 35 cycles of incubation for 30 s at 94 °C, 30 s at 60 °C, and 30 s at 72 °C, and completed with a final extension incubation at 72 °C for 5 min. Amplicons were then sequenced in both directions.

### 2.5. Genotyping Using PARMs-PCR and Mass Spectrometry

All the 225 sheep DNA samples were genotyped for individual SNPs using a PARMS–polymerase chain reaction (PARMS-PCR) approach, following the procedure described by Sun et al. [14]. For each SNP, three primers were designed, a common forward primer and two reverse primers that contained either FAM or HEX fluorescent sequences at the 5’ end. The sequence and fluorescent signal type information are shown in Table 2, and they were synthesized by Gentides Technology Co., Ltd. (Wuhan, China).

The PARMS-PCR amplification thermal cycle was pre-denaturation at 94 °C for 15 min, denaturation at 94 °C for 20 s, and a 65 °C (−0.7 °C per cycle) annealing and extension step for 1 min, for 10 cycles. This was followed by denaturation at 94 °C for 20 s and a 57 °C annealing and extension step for 1 min, for 35 cycles. The fluorescence was detected with a TECAN Infinite F200 Enzyme Labeler (AiYan Biotechnology Co., Ltd., Shanghai, China) and then parsed and converted into fluorescence signals using the online software SNP Decoder (http://www.snpway.com/snpdecoder/; accessed on 5 October 2023), which outputted the genotype results. Unique haplotypes were prepared and then sequenced by Gentides Technology Co., Ltd. (Wuhan, China).

### 2.6. RT-qPCR

The sheep *KRT84 mRNA* sequence (XM_004006329.5) was used to design a pair of PCR primers to amplify a fragment of 3′-UTR from the cDNA. The β-actin gene (*ACTB*) (NM_001009784) was used as a reference to calibrate the level of gene expression. These primer sequences are shown in Table 1. Approximately 0.4 μg of total RNA was used for reverse transcription and complementary DNA (cDNA) was prepared using a PrimeScript^TM^ RT kit (Nanjing Vazyme Biotechnology Co., Ltd., Nanjing, China) according to the kit instructions. The qPCR reactions were performed using a Biosystems QuantStudioR 6 Flex (Thermo Fisher Scientific Inc, Waltham, MA, USA) platform with the annealing temperatures described in Table 1, the cDNA from above as the template, and SYBR Green Pro Taq HS qPCR kits (Hunan Accurate Biotechnology, Changsha, China). In 20 μL reactions, the thermal cycle parameters were as follows: initial denaturation at 95 °C for 10 min, followed by 45 cycles of 15 s at 95 °C and 60 °C annealing and extension for 1 min. The cDNAs were quantified in triplicate, with four technical replicates.

### 2.7. Immunofluorescence Analysis

Diao’s immunofluorescence method was used in this experiment [15]. The primary antibody used in the experiment was K84 (TD10116, rabbit antibody) and the secondary antibody was CY3 (GB21303, goat anti-rabbit IgG), provided by Servicebio Technology Co., Ltd. (Wuhan, China). The immunofluorescence results were scanned and imaged using a PANNORAMIC panoramic slice scanner (3DHISTECH, Budapest, Hungary). The outputted scan files were opened with the CaseViewer 2.2 software using 200× magnification. The integral optical density (IOD) and the corresponding positive pixel area (area) of three fields of view for each slice were measured separately using Image-Pro Plus 6.0 software (Media Cybernetics Inc., Rockville, MD, USA) to calculate the average optical density (AOD) using IOD/Area = AOD. The scoring was performed on three fields of view for the corneum, dermal papilla, sebaceous gland, outer root sheath, inner root sheath, and hair matrix for each slice. AOD was used to indicate the degree of positive intensity.

### 2.8. Statistical Analyses

The genotype and haplotype frequencies of the *KRT84* SNPs were counted using Excel 2019 software, and an online calculator (http://www.msrcall.com/Gdicall.aspx; accessed on 12 October 2023) was used to calculate homozygosity (Ho), heterozygosity (He), polymorphism information content (PIC), and effective allele numbers (Ne). The Hardy–Weinberg equilibrium of the haplotypes was tested using the chi-square test in the online calculator. The SNP chain imbalance analysis and haplotype analysis were performed using Haploview 4.2 software, after eliminating the results from sheep that could not be typed.

General linear models (IBM SPSS 26.0) were used to analyse the association of the different haplotypes revealed with the measured wool traits MFD, CVFD, MSL, MFC, CF, and MSS in the Gansu Alpine Fine-wool sheep. The model was yijk = µ + Hi + eijk, where yijk indicates the trait phenotype value, µ denotes the overall mean, Hi indicates a haplotype combination, and eijk denotes the random error. The results were output as mean ± standard error (S.E.), with *p* < 0.05 as the criteria for accepting significant differences.

The RT-qPCR results were calculated in Excel using the 2^−ΔΔCT^ [16] method and correlation analysis with wool trait data.

## 3. Results

### 3.1. Localization of K84 mRNA in the Skin of Gansu Alpine Fine-Wool Lambs

**Figure 1:**
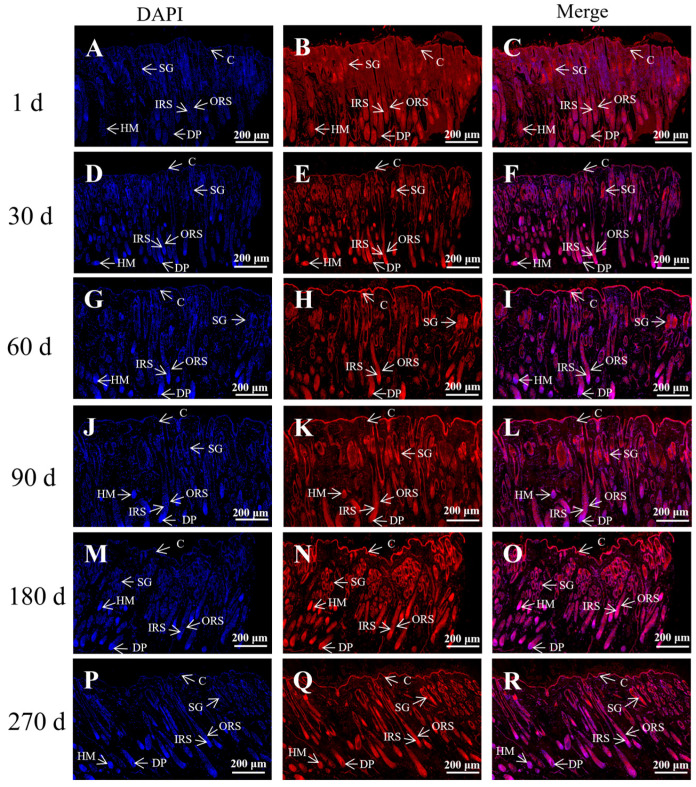
Immunofluorescence staining for K84 in the skin of Gansu Alpine Fine-wool sheep at different times. Immunofluorescence staining of K84 (5×). (**A**–**C**) 1d; (**D**–**F**) 30 d; (**G**–**I**) 60 d; (**J**–**L**) 90 d; (**M**–**O**) 180 d; (**P**–**R**) 270 d. The blue tissue in the figure is the DAPI-labelled nuclear fluorescence staining and the red tissue is the fluorescence staining of K84. C: corneum; DP: dermal papilla; SG: sebaceous gland; ORS: outer root sheath; IRS: inner root sheath; HM: hair matrix.

The immunofluorescence analysis of the distribution of K84 in the skin tissues of Gansu Alpine Fine-wool sheep at different lamb ages (Figure 1) revealed that K84 was highly expressed in hair follicles such as the inner root sheath (IRS), outer root sheath (ORS), and hair medulla (HM) and at all six lamb ages. The dermal papilla at 1, 60, and 90 days of age had strong expression of K84; and skin tissues at 30, 180, and 270 days of age had strong K84 expression.

### 3.2. K84 Levels in the Skin of Gansu Alpine Fine-Wool Sheep at Different Lamb Ages

**Figure 2:**
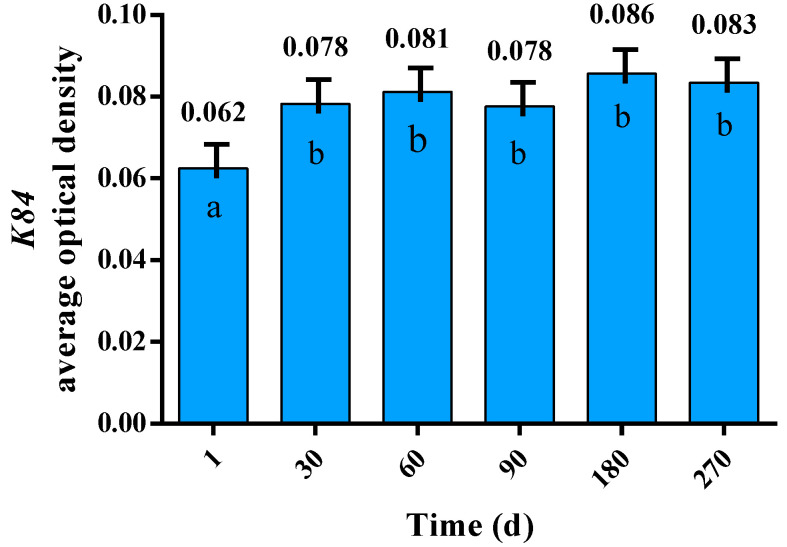
Levels of K84 in the skin of Gansu Alpine Fine-wool sheep at different times. The data are expressed as means ± S.E. Different letters indicate significant differences between lambs of differing age (*p* < 0.05).

The average optical density of the tissue sections is revealed in Figure 2. The protein K84 was found at different levels in the skin of lambs of differing age, with an overall trend towards an increase. The amount of K84 was significantly higher at 30, 60, 90, 180, and 270 days, when compared to the level at birth (Day 1; *p* < 0.05).

### 3.3. SNP Detection and Genotyping

**Figure 3:**
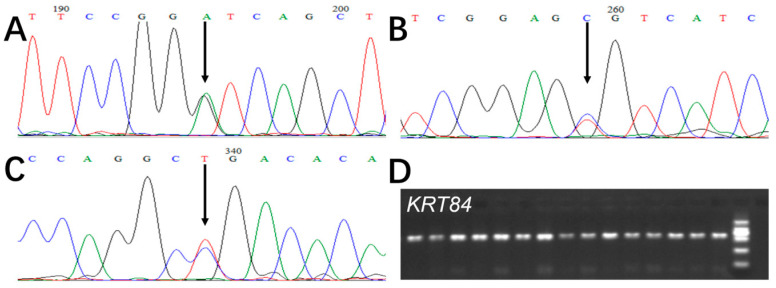
PCR amplification and sequencing of *KRT84* in Gansu Alpine Fine-wool sheep. (**A**) c.97A/G, (**B**) c.162C/T, (**C**) c.286G/A (noting that the overlapping peaks indicated by arrows in A, B, and C are SNPs and that the numbering on the electropherograms reflects the location in the sequencing reaction and not the position of the SNP in the gene). Sequences in A and B are from the forward strand whereas the sequence in C is from the reverse strand. (**D**) D illustrates the PCR amplification of *KRT84*.

In Figure 3, the DNA sequencing of exon 1 amplicons revealed three SNPs in Gansu Alpine Fine-wool sheep, and these were named c.97A/G, c.162C/T, and c.286G/A, according to their position in the gene.

**Figure 4:**
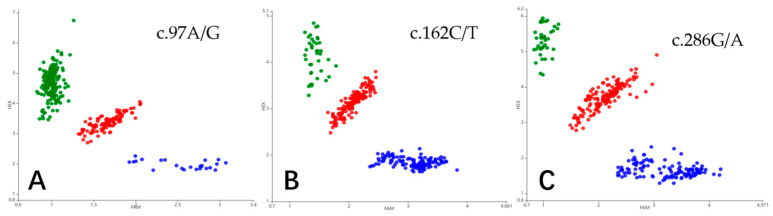
Genotyping of *KRT84* in Gansu Alpine Fine-wool sheep using PARMS analysis. (**A**) The red, green, and blue dots indicate GA, GG, and AA, respectively; (**B**) The red, green, and blue indicate CT, TT, and CC, respectively; (**C**) The red, green, and blue dots indicate GA, GG, and AA, respectively.

In Figure 4, the PARMS labelling assay produced scatterplots, where the horizontal coordinate is the FAM fluorescence value, and the vertical coordinate is the HEX fluorescence value. The heterozygous genotypes are in the middle of the scatterplot (indicated with red dots), while the homozygous sheep are located at the edges of the scatterplot and are indicated by blue and green dots.

### 3.4. Analysis of the Sequence Variation in KRT84

The polymorphic information content (PIC), homozygosity (Ho), heterozygosity (HE), and number of effective alleles (Ne) of *KRT84* are shown in Table 3. The PICs of the three SNPs (c.97A/G, c.162C/T, and c.286G/A) were 0.3577, 0.6956, and 0.6965, respectively. All the SNPs were in Hardy–Weinberg equilibrium in this population (*p* > 0.05), with this suggesting that these Gansu Alpine Fine-wool sheep were not inbred or subject to some form of selection pressure for *KRT84* variation.

The SNP haplotype frequencies and genotype frequencies are revealed in Table 4. For c.97A/G, G was the most common nucleotide, with a frequency of 0.757, and GG was the most common genotype, with a genotype frequency of 0.589. For c.162C/T, C was the most common nucleotide with a frequency of 0.664 and CC was the most common genotype, with a frequency of 0.444. At c.286G/A, G was the more common nucleotide, with a frequency of 0.664, and GA was the most common genotype, with a frequency of 0.449.

### 3.5. Haplotype Construction for the KRT84 Exon 1 SNPs and Association Analyses between These Haplotypes and Wool Traits

**Figure 5:**
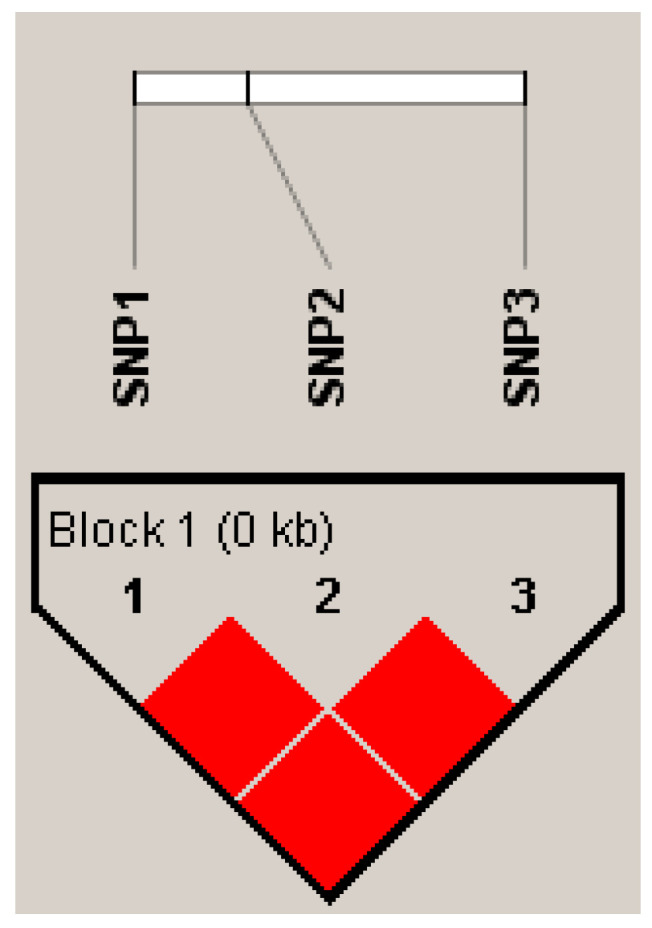
Linkage disequilibrium analysis of the *KRT84* SNPs in Gansu Alpine Fine-wool sheep. The red square in the figure has an R^2^ value of 1, suggesting a strongly interlocking state.

Linkage disequilibrium analysis was performed on the three SNPs in *KRT84*. Haplotypes were constructed for the three SNPs, with reporting of those haplotype combinations (diplotypes). This allowed the construction of a haplotype block (Figure 5), and the SNPs were in a strongly interlocking state (R^2^ = 1), with three haplotypes detected that were named *H1*-*H3* (Table 5). The haplotype with the highest frequency in the Gansu Alpine Fine-wool sheep was *H1*. Combinatorial analysis of the three haplotypes yielded a total of six diplotypes with frequencies greater than 0.05 (Table 5), with the *H1H2* diplotype having the highest frequency (28.25%).

Association analyses of these six diplotypes with wool traits (Table 6) suggested an effect of *KRT84* variation on MFD, CVFD, MSL, MFC, CF, and MSS at a significance level of *p* < 0.05.

The MFD of the homozygous *H1H1* sheep was lower than the *H3H3* or *H1H3* sheep (*p* < 0.05), while the CVFD of the homozygous *H1H1* sheep was also lower than the *H3H3* or *H1H3* sheep (*p* < 0.05). The MSL of the homozygous *H1H1* sheep was greater than the *H3H3* or *H1H3* sheep (*p* < 0.05), yet the MFC of the homozygous *H2H2* sheep was higher than *H1H1*, *H1H2*, *H1H3,* and *H2H3* (*p* < 0.05). The CF of *H1H1* sheep was higher than *H1H3* and *H3H3* (*p* < 0.05), while the MSS of the homozygous *H3H3* sheep was higher than the *H1H1*, *H1H2*, *H1H3,* and *H2H3* (*p* < 0.05). Diplotype did not appear to affect FDSD (*p* > 0.05).

**Figure 6:**
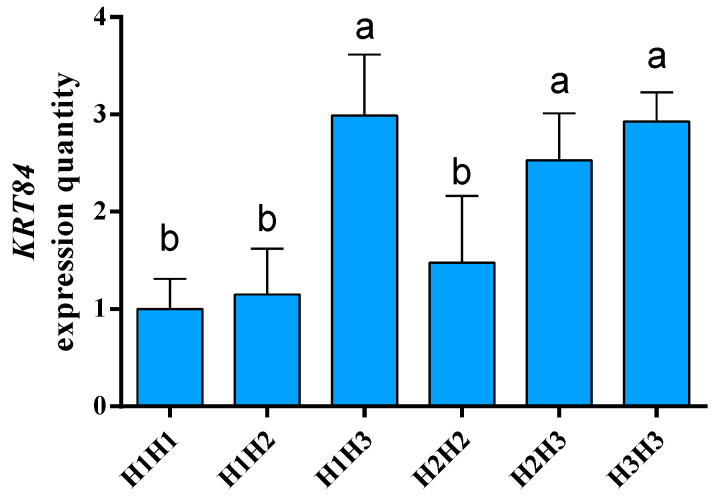
The mRNA levels of *KRT84* in the skin of Gansu Alpine Fine-wool sheep. The β-actin gene (*ACTB*) was used as a reference gene to calibrate the level of gene expression. The genotype expression levels in skin are presented as means ± S.E. and significant differences (*p* < 0.05) are denoted by different letters.

Quantitative fluorescence analysis (Figure 6) suggested that the *KRT84* mRNA levels of *H1H3*, *H2H3,* and *H3H3* were higher than the levels of *H1H1*, *H1H2,* and *H2H2*.

## 4. Discussion

### 4.1. Variation in KRT84

It is not unprecedented for ovine keratin genes to contain nucleotide sequence variation, with evidence for this being presented in analyses of the whole genome and studies of individual genes such as *KRT31* [17], *KRT33A* [18], and *KRT83* [19]. Analysis of *KRT84* in Ensembl (release 110, 2023) reveals a sequence in the Rambouillet v1.0 genome construct CM008474.1 (a fine wool sheep breed), identified as ENSOARG00020013379 (GenBank GENE ID: 10056798), which is near another keratin gene *KRT82* (EN-SOART00020020319). The *KRT84* sequence has nine exons, with six containing SNPs. Some SNPs in exons 1, 2, 7, and 9 are non-synonymous, with splice region variants also described upstream of exons 6 and 9. Exon 1 encodes the head region of *KRT84*, while exons 2–8 encode the intermediate filament rod domain. The identified coding sequence is 1782 nucleotides in length with the ATG start site in exon 1 and a predicted length of 593 amino acids.

The SNPs c.97A/G and c.286G/T are both missense, and while c.97A/G (p.Ile33Val) is a conservative change, c.286G/T is far less so, with glycine putatively being substituted with cysteine. In keratins, cysteine is known to cross-link with both other keratins and the keratin-associated proteins; hence, this SNP potentially changes the ability of K84 to interact with other proteins in the wool fibre, and thus possibly the wool fibre structure and function. In this respect, if expressed, this amino change would be in the head region of K84, a region that is important in the formation of the keratin dimers and tetramers that arrange into protofilaments. In the report of Harland et al. [20], fibers from Romney sheep were subjected to stretching or to their breaking point under wet or dry conditions to detect disulfide bonds breakage. Many of the identified cysteine residues were located close to the terminal ends of the keratins (head or tail domains), where they likely affect keratin-associated protein interactions and keratin interactions.

### 4.2. Association Analysis between Haplotypes of KRT84 and Wool Traits for Gansu Alpine Fine-Wool Sheep Wool

The discovery of variation in wool keratin genes has led to the question of whether it affects the growth and development of wool fibres. In that respect, nucleotide sequence variation in *KRT31* has been associated with variation in greasy fleece weight (GFW), clean fleece weight (CFW), and MSL in Merino-cross sheep [17], while variation in *KRT33A* has been associated with variation in fibre crimp frequency, MFC, wool bulk, fleece weight, MSL, and washing yield (CFW/GFW × 100%) in Perendale sheep [18]. Within half-sib families of Merino and Merino-cross sheep, variation in *KRT33A* was associated with FDSD, MSS, and wool colour in one family, and with MSL in another family [21]. In 2017 Chai et al. [19] revealed variation in *KRT83* to be associated with variation in FDSD, CVFD, MFD, MFC, PF, and a decrease in washing yield, and in 2022, Chai et al. [22] reported that variation in the promotor region of *KRT34* was associated with MFD, FDSD, and MSL.

The *KRT84* SNPs in Gansu Alpine Fine-wool sheep described here were also associated with several traits, including wool MFD, CVFD, MSL, MFC, CF, and MSS. Many of these are important wool traits, not least MFD, which typically is the key determinant of the value proposition for wool. This indicates that the other traits like CVFD, MSL, MSS, FDSD, and MFC all can affect wool value too. In respect to MFC, and given there was nearly an 8 degree difference in curvature difference between *H1H1* and *H2H2* sheep, research with mice has revealed that two missense mutations in the first exon of mouse *KRT71* lead to the production of wavy hair [23] and Kuramoto et al. [24] demonstrated that in rats, the Rex mutation in *KRT71* (a 7 bp deletion at the splice acceptor site of intron 1) causes curly hair.

While the associations between variation in keratin genes and wool fibres traits are noteworthy, it should also be noted that several studies have linked variation in the keratin-associated proteins with variation in wool traits. These associations are in part summarized in Gong et al. [25]. It must also be remembered that wool growth is influenced by dietary factors, for example with Coetzee et al. [26] illustrating how infusion of the amino acids methionine, lysine, and rumen-protected methionine derivates affect wool growth rate. Future studies could therefore investigate the relationship, if it exists, between specific dietary factors or regimes and the expression of the keratin genes.

### 4.3. Localization of KRT84 Gene in the Skin of Gansu Alpine Fine-Wool Sheep at Different Lamb Ages

In this experiment, the presence of the wool keratin protein K84 was investigated in the skin and wool follicles at different stages of post-natal growth, using a K84 specific antibody and immunofluorescent detection methods. This revealed that the protein was present in the follicle IRS, the ORS, and the HM over the different ages of the lambs. This contrasts with some of the findings of Yu et al. [27]. They reported that *KRT84* could be amplified from total sheep skin RNA from the Wiltshire breed (a shedding sheep breed) using primers designed from the bovine *KRT84* ortholog, but they only obtained a weak signal using an RT-PCR approach. Analysis of where the gene appeared to be expressed suggested expression in the fibre cuticle, the medulla, and what is called the ‘brush end’. The function of the IRS may be to determine the shape of the fibre during hair growth, and its growth characteristics and defects may affect the shape of the hair, resulting in hairs with curved traits. Yu et al. [27] revealed that the *KRT32*, *KRT35* and *KRT85* genes were expressed in both the IRS and fibre cuticle, while *KRT36* and *KRT87* were expressed only in cortical layers. Only *KRT34* and *KRT36* had high levels of expression in the HM, but *KRT82* was widely expressed from the middle of the hair bulb to the upper part of the cuticle.

In a study of Merino sheep wool, Hynd et al. [28] found that different growth rates of cortical cells in the upper part of the wool bulb resulted in a curved trait. It was found that some keratin genes were asymmetrically expressed, including *KRT32*, *KRT35,* and *KRT85*, with these genes expressed in the cuticle and fibre cortex and where they appear to be involved in the differentiation of the cortex. This asymmetry was not obvious for *KRT35* and *KRT85* in the work of Yu et al. [27], but *KRT32* may have been asymmetrically distributed and *KRT38* appeared to be asymmetrically distributed in the cortex. While the distribution and location of *KRT* expression appears inconsistent, which may reflect differences between different animals and breeds, Hynd et al. [28] have suggested that the associations between cortical properties and fibre crimp are not consistent and that they therefore may not reflect the underlying causation of fibre curvature. They instead suggested that variations in fibre crimp could be accounted for by quantitative differences in both the degree of mitotic asymmetry in follicle bulbs and the distance from the bulb to the point at which keratinization is completed.

Based on the effect of *KRT84* variation on several wool traits in Gansu Alpine Fine-wool sheep in this study, it is hypothesized that the gene plays an important role in the development of the wool follicles and therefore the fibre, where it appears to affect wool traits. However, further studies on the relationship between *KRT84* (and other *KRTs* and keratin-associated protein genes) and wool traits are needed to fully understand how these genes affect fibre properties, which ultimately may enable the development of hair disease therapies, keratin-based cosmetic ingredients, and for sheep selection to improve wool quality. In this respect, improving the quality of the wool produced by Gansu Alpine Fine-wool sheep would not only better meet the diversified needs of markets but also likely increase the income of farmers.

## 5. Conclusions

Expression of the *KRT84* gene appears to vary during post-natal growth (days 0 to 270) and vary across different locations in the skin. This lays a foundation for more research into the level and location of expression of other keratin and keratin-associated protein genes. The discovery of associations between *KRT84* diplotypes and wool traits suggests that *KRT84* affects MFD, MSL, MFC, CF and MSS, with sheep with the *H3H3* diplotype appearing to have increased MFD, CVFD and MSS and reduced MSL and CF compared to *H1H1* sheep. The gene *KRT84* might therefore be useful as a gene-marker for breeding Gansu Alpine Fine-wool sheep and other breeds too, if that same variation is found in them.

## Figures and Tables

**Table 1:** Primer sequences and annealing temperatures used for PCR and RT-qPCR.

Gene	Primer Sequences (5′–3′)	Size (bp)	Annealing Temperature (°C)	Use
*KRT84*	F: CCATCATGTCTTGCCGCTCCT	587	60	PCR
	R: ACACCCCAGTGCATTTAGCTC			
*KRT84*	F: ACGATCTTCAGTGGCCCAAG	104	60	RT-qPCR
	R: GGGGTTAAACCAACACCCCA			
*ACTB*	F: AGCCTTCCTTCCTGGGCATGGA	113	60	RT-qPCR
	R: GGACAGCACCGTGTTGGCGTAGA			

**Table 2:** PARMS-PCR primer sequences and fluorescent signal types.

SNP	Primer	Primer Sequences (5′–3′)	Fluorescent Signal	Genotype
c.97A/G	1Rt	GAAGGTGACCAAGTTCATGCTCCTGCAGGAGACAGAGCTGAT	FAM	A
	1Rc	GAAGGTCGGAGTCAACGGATTCCTGCAGGAGACAGAGCTGAC	HEX	G
	1F	CCCCTCAGAACCTGAACCG	/	/
c.162C/T	2Rg	GAAGGTGACCAAGTTCATGCTACGATCCAAAGCTGATGACG	FAM	C
	2Ra	GAAGGTCGGAGTCAACGGATTCACGATCCAAAGCTGATGACA	HEX	T
	2F	CCCCTCAGAACCTGAACCG	/	/
c.286G/A	3Rc	GAAGGTGACCAAGTTCATGCTGGCTGCAGAAGCCATAGCC	FAM	G
	3Rt	GAAGGTCGGAGTCAACGGATTGGGCTGCAGAAGCCATAGCT	HEX	A
	3F	GGGGTTTAGAGCCAGAAGTGG	/	/

**Table 3:** The population genetic diversity of the three SNPs in exon 1 of ovine *KRT84*.

SNP	Ho	He	PIC	Ne	H
c.97A/G	0.5335	0.4665	0.3577	1.8743	0.6592
c.162C/T	0.2738	0.7262	0.6956	3.6528	0.7173
c.286G/A	0.2735	0.7265	0.6965	3.6566	0.7156

Ho: homozygosity; He: heterozygosity; PIC: polymorphic information content; Ne: number of effective alleles; H: Hardy–Weinberg equilibrium; PIC < 0.25 low polymorphism, 0.25 < PIC < 0.5 for moderate polymorphism, PIC > 0.5 for highly polymorphic.

**Table 4:** SNP genotype frequencies and nucleotide frequencies for exon 1 of *KRT84*.

SNP	SNP Genotype Frequency (Number)			Nucleotide Frequency		X^2^	*p*
c.97A/G	GA	GG	AA	G	A	0.8304	0.660
	0.335(75)	0.589(132)	0.076(17)	0.757	0.243		
c.162C/T	CT	CC	TT	C	T	0.0181	0.991
	0.440(99)	0.444(100)	0.116(26)	0.664	0.336		
c.286G/A	GA	GG	AA	G	A	0.0035	0.006
	0.449(101)	0.440(99)	0.111(25)	0.664	0.336		

Note: The figures in parentheses are the number of individuals that were genotyped.

**Table 5:** Frequency of haplotypes and diplotypes for the SNPs in exon 1 of *KRT84*.

Haplotype	c.97A/G	c.162C/T	c.286G/A	Frequency (%)	Diplotype	Frequency (%)
*H1*	G	C	G	42.30	*H1H1*	19.28
*H2*	G	T	A	33.50	*H1H2*	28.25
*H3*	A	C	G	24.20	*H1H3*	17.49
					*H2H2*	11.21
					*H2H3*	16.14
					*H3H3*	7.62

**Table 6:** Relationship between *KRT84* haplotypes and selected key wool traits.

Diplotype	MFD (Microns)	FDSD (Microns)	CVFD	MSL (mm)	MFC (°/mm)	CF (%)	MSS (cN/dT)
*H1 H1*	21.4 ± 2.56 ^b^	5.4 ± 1.12	24.9 ± 3.36 ^b^	77.4 ± 15.05 ^a^	103.9 ± 12.48 ^b^	91.4 ± 9.93 ^a^	14.0 ± 6.75 ^b^
*H1 H2*	22.1 ± 2.95 ^ab^	5.5 ± 1.06	25.0 ± 2.99 ^ab^	74.0 ± 11.80 ^ab^	105.6 ± 9.94 ^b^	88.6 ± 11.18 ^ab^	13.6 ± 5.22 ^b^
*H1 H3*	22.9 ± 2.81 ^a^	5.8 ± 1.13	25.4 ± 2.95 ^a^	70.9 ± 14.87 ^b^	104.7 ± 12.54 ^b^	85.9 ± 11.72 ^b^	13.7 ± 5.05 ^b^
*H2 H2*	22.3 ± 3.20 ^ab^	5.5 ± 0.94	24.6 ± 2.60 ^b^	68.2 ± 13.90 ^b^	111.5 ± 11.15 ^a^	87.6 ± 11.62 ^ab^	15.3 ± 6.15 ^ab^
*H2 H3*	22.4 ± 2.83 ^ab^	5.6 ± 0.92	25.0 ± 2.42 ^ab^	71.3 ± 13.56 ^ab^	104.9 ± 11.15 ^b^	87.5 ± 11.98 ^ab^	14.1 ± 6.60 ^b^
*H3 H3*	23.2 ± 3.63 ^a^	6.0 ± 0.78	26.3 ± 3.43 ^a^	71.9 ± 16.94 ^b^	106.5 ± 13.71 ^ab^	82.4 ± 15.60 ^b^	17.5 ± 6.60 ^a^

Different letters in the columns indicate significant differences (*p* < 0.05). The results were expressed as means ± standard errors (S.E.). MFD: mean fibre diameter, FDSD: fibre diameter standard deviation, CVFD: coefficient of variation of fibre diameter, MSL: mean staple length, MFC: mean fibre curvature, CF: comfort factor, MSS: mean staple strength.

## Data Availability

The authors affirm that all data necessary for confirming the conclusions of the article are present within the article, figures, and tables.
